# Estrategia de atención primaria de salud y su impacto en las hospitalizaciones evitables por condiciones sensibles a la atención ambulatoria, Paraguay, 2000-2017

**DOI:** 10.26633/RPSP.2019.69

**Published:** 2019-08-22

**Authors:** María José Lerea, Juan Edgar Tullo, Pedro López

**Affiliations:** 1 Dirección de Análisis de la Información en Salud/Dirección General de Información Estratégica en Salud Dirección de Análisis de la Información en Salud/Dirección General de Información Estratégica en Salud Ministerio de Salud Pública y Bienestar Social Paraguay Dirección de Análisis de la Información en Salud/Dirección General de Información Estratégica en Salud, Ministerio de Salud Pública y Bienestar Social, Paraguay.; 2 Organización Panamericana de la Salud Organización Panamericana de la Salud Paraguay Organización Panamericana de la Salud, Paraguay.

**Keywords:** Hospitalizaciones, atención primaria de salud, Paraguay, Hospitalization, primary health care, Paraguay, Hospitalização, atenção primária à saúde, Paraguai

## Abstract

**Objetivo.:**

Explorar la influencia que ha tenido la implementación y fortalecimiento de la estrategia de atención primaria (APS) sobre las hospitalizaciones por condiciones sensibles al cuidado ambulatorio (HCSCA).

**Métodos.:**

Estudio observacional descriptivo y transversal, que consideró datos correspondientes a los registros de internaciones de hospitales públicos de todo el país en el período 2000-2017. Se considera el año 2009 como punto de inicio de la estrategia de APS. Para definir los diagnósticos de las HCSCA se utilizaron los propuestos por la Organización Panamericana de la Salud/Organización Mundial de la Salud. Se realizó el análisis de su comportamiento considerando dos etapas: 2000-2008 y 2009-2017.

**Resultados.:**

Las HCSCA mostraron una tendencia a la disminución, se halló una diferencia de 6,75% entre los años finales de cada etapa. La disminución fue mayor en la medida que se amplió la cobertura de APS. Las principales causas de HCSCA fueron la neumonía y la enfermedad diarreica aguda. Las internaciones por enfermedades crónicas disminuyeron en su mayoría: por el contrario, los ingresos por enfermedades infecciosas aumentaron.

**Conclusiones.:**

La cobertura y acceso poblacional con unidades de salud familiar puede limitar el impacto de la APS en las HCSCA. Se verifica una disminución de las HCSCA luego de la implementación de la APS en Paraguay. Las internaciones por enfermedades crónicas muestran mejor resultado que las internaciones por enfermedades infecciosas.

## Abstract (en)

**Objective.:**

Explore the effect of the implementation and strengthening of the primary care strategy (PHC) on hospitalizations for ambulatory care-sensitive conditions (HACSC).

**Methods.:**

Descriptive cross-sectional observational study that considered the data from public hospital admissions records for the entire country for the period 2000-2017. The starting point of the PHC strategy is considered to be 2009. The diagnoses proposed by the Pan American Health Organization/World Health Organization were used in defining the HACSC diagnoses. The analysis of HACSC behavior considered two stages: 2000-2008 and 2009-2017.

**Results.:**

HACSC trended downward, with a difference of 6.75% between the final years of each stage. The greater the PHC coverage, the larger the reduction. The leading causes of HACSC were pneumonia and acute diarrheal disease. Admissions for chronic diseases largely fell, while admissions for infectious diseases rose.

**Conclusions.:**

The degree of population coverage and access to family health units can limit the impact of PHC on HACSC. A reduction in HACSC was confirmed after PHC implementation in Paraguay. The results for admissions for chronic diseases are better than those for infectious diseases.

## RESUMO

**Objetivo.:**

Explorar a influência da implementação e fortalecimento da estratégia de atenção primária à saúde (APS) nas internações hospitalares por causas sensíveis à atenção ambulatorial.

**Métodos.:**

Estudo observacional descritivo transversal realizado com dados de registros de internações em hospitais públicos de todo o país no período entre 2000 e 2017. O ano de 2009 é considerado o ponto inicial da estratégia de APS no Paraguai. Os diagnósticos das internações hospitalares por causas sensíveis à atenção ambulatorial foram definidos segundo os critérios propostos pela Organização Pan-Americana da Saúde/Organização Mundial da Saúde (OPAS/OMS). A análise foi realizada em duas fases distintas: 2000–2008 e 2009–2017.

**Resultados.:**

Verificou-se uma tendência de redução das internações hospitalares por causas sensíveis à atenção ambulatorial, com uma diferença de 6,75% entre os anos finais de cada fase. A redução foi maior à medida que se ampliou a cobertura de APS. As principais causas de internações hospitalares foram pneumonia e doença diarreica aguda. Houve redução das internações por doenças crônicas na sua maioria e, em contraste, houve aumento das internações por doenças infecciosas.

**Conclusões.:**

A cobertura e o acesso da população a unidades de saúde da família são fatores limitantes ao impacto da APS nas internações hospitalares por causas sensíveis à atenção ambulatorial. Verifica-se uma redução das internações hospitalares após a implementação da APS no Paraguai. Os resultados são melhores para internações por doenças crônicas em comparação às internações por doenças infecciosas.

El primer nivel de atención constituye la puerta de entrada de la población al sistema de salud. Se caracteriza por contar con establecimientos de baja complejidad que resuelven aproximadamente 85% de los problemas prevalentes. La atención primaria de salud (APS) se define como: “la asistencia sanitaria esencial, basada en métodos y tecnologías prácticos, científicamente fundados y socialmente aceptables, puesta al alcance de todos los individuos de la comunidad, mediante su plena participación y a un costo que la comunidad y el país puedan soportar en todas y cada una de las etapas de su desarrollo, con espíritu de autorresponsabilidad y autodeterminación” (1).

Un modelo adecuado de APS debe proveer mejores resultados que otras alternativas de políticas de salud mediante la prevención temprana, el diagnóstico oportuno y el seguimiento de los pacientes en sus problemas de salud, tanto agudos como crónicos (2). Además, un sistema de salud organizado a partir de la APS resulta más costo-efectivo que uno direccionado para el acceso a la atención especializada. Por esa razón, existe el consenso de que el fortalecimiento de la APS es la estrategia más adecuada para la reforma del sector salud, en especial con el crecimiento de la incidencia de enfermedades crónicas no transmisibles (3).

En congruencia con esto, en años recientes, las estrategias para lograr mejores resultados en salud de muchos países de la Región (Argentina, Brasil Chile, Costa Rica, Ecuador y Paraguay, entre otros) consisten en ampliaciones de cobertura en el primer nivel de atención mediante la expansión de equipos básicos de salud integrados con la organización en red de los cuidados más complejos. La tarea de monitorización y evaluación de dichas estrategias resulta difícil, una vez que los resultados en salud son determinados por múltiples caminos causales y la vida de un adulto representa un largo período de exposición a factores de riesgo de diversa índole (4).

En la práctica cotidiana, se constatan ineficiencias en el sistema de salud que ocasionan la hospitalización por problemas de salud que no se agravarían ni requerirían hospitalización si la atención fuese oportuna y efectiva (5). En este sentido, se propusieron las hospitalizaciones evitables por condiciones sensibles al cuidado ambulatorio (HCSCA) como indicadores de calidad de la APS (6). Esta elección está respaldada por evidencia científica en la extensa bibliografía internacional (4). En ese contexto, las HCSCA pueden ser un indicador indirecto de la capacidad de resolución de la APS, de la efectividad, de la eficiencia, de la calidad de los servicios y de la reducción de los costos hospitalarios (7). Este concepto se desarrolló en Estados Unidos de América a mediados de los años ochenta para analizar el acceso de la población a la atención médica, y a finales de los años noventa lo propuso el Servicio Nacional de Salud británico para el análisis de la calidad de la APS (8).

En la actualidad, varios organismos como la Organización Panamericana de la Salud (OPS) (9) e instituciones lo usan para evaluar los servicios de salud, aunque esa medida rara vez ha sido utilizada para estudiar el desempeño del sistema de salud en países de ingresos bajos y medios (10). Se espera que una mejor respuesta de los servicios de APS implique una disminución de los ingresos hospitalarios. Este concepto es el propuesto para evaluar el funcionamiento del primer nivel de atención, en el que se toman como referencia las hospitalizaciones debidas a causas que podrían haber sido prevenidas o evitadas mediante una intervención adecuada de la APS, para cada problema de salud (11).

Desde el año 2009, Paraguay apuesta por un sistema de salud basado en la APS, con el fin de resolver las brechas existentes y garantizar el acceso y cobertura universal de salud. La nueva estrategia de APS se implementa con la creación de unidades de salud de la familia (USF), que es la estructura física y funcional en la que se desarrolla una parte importante de la estrategia de forma coordinada, integral, continuada, permanente y con base en el trabajo de un equipo de salud familiar (ESF). Este equipo asume la responsabilidad sanitaria y social de la atención de una comunidad definida desde los puntos de vista demográfico y geográfico, y en sus actividades han de estar contemplados tantos los aspectos de prevención de la enfermedad y promoción de la salud como la prestación de servicios asistenciales (12). Hasta el año 2017, había en el país 801 USF, con las que alcanzaba una cobertura de 32% de la población (13).

Por otra parte, el Ministerio de Salud Pública y Bienestar Social (MSPBS) cuenta con 215 instituciones con capacidad de internación, que disponen de 5 372 camas. La tasa de egreso anual en estas instituciones es de 39,6 por cada 1000 habitantes (14).

El MSPBS recolecta los datos relacionados con las hospitalizaciones en todos los establecimientos del sector público: por lo tanto, se dispone de la información necesaria para realizar la medición del indicador HCSCA con el objetivo de explorar la influencia que ha tenido la implementación y fortalecimiento de la estrategia de APS sobre él.

## MATERIALES Y MÉTODOS

Se realizó un estudio descriptivo y transversal con base en los datos del Subsistema de Egreso Hospitalario (SEGHOSP) como fuente de información y que contiene los registros de todos los hospitales públicos del país que cuentan con internación. Se consideró para el estudio un período de 18 años (desde el año 2000 hasta el año 2017).

Se definen como HCSCA aquellos diagnósticos potencialmente prevenibles con una adecuada implementación de la estrategia de APS. Para definir el listado de diagnósticos de HCSCA se tomaron como referencia los propuestos por la OPS en el *Compendio de indicadores de impacto y resultados intermedios* y están clasificados dentro de la Clasificación Internacional de Enfermedades, Décima revisión (CIE-10) (9).

Según la Encuesta Permanente de Hogares realizada por la Dirección General de Estadísticas Encuestas y Censo en el año 2017, solo 19,3% de la población cuenta con seguro médico de IPS (Instituto de Previsión Social) y 7,5% tiene otro tipo de seguro, lo que totaliza 26,8% de la población paraguaya que cuenta con seguro médico. El resto (73,04%) declara que no cuenta con seguro médico, por lo que la mayoría de esa población accede a los servicios del MSPBS.

La población estuvo compuesta por 3 084 398 registros de internaciones que corresponden al total de egresos hospitalarios generados en el período. La muestra estuvo constituida por aquellos registros hospitalarios cuyos diagnósticos al egreso correspondió a una HCSCA, con un total de 539 950 registros.

Se realizó el análisis considerando dos etapas acumuladas: la etapa 1(2000-2008) antes de la implementación de la APS, y la etapa 2 (2009-2017), posterior a la implementación. Se confeccionaron figuras y cuadros estadísticos que permitieron visualizar mejor la información.

Se respetaron los postulados éticos de la investigación. No fue necesario solicitar consentimiento informado a ningún paciente por trabajar con datos estadísticos. La información no será utilizada fuera del marco de la investigación.

## RESULTADOS

Del total de las hospitalizaciones, 18% corresponde a las HCSCA. En la primera etapa, este porcentaje fue de 17,6%; en la segunda etapa fue de 17,5%, lo que representa una reducción del porcentaje (0,1%) de internaciones en la segunda etapa.

La distribución según diagnósticos muestra que la neumonía y la enfermedad diarreica aguda (EDA) son las causas más frecuentes y, en ambas etapas, ocupan, entre los dos, aproximadamente 50% de las HCSCA. Se detecta disminución de las internaciones por enfermedades crónicas, excepto para el infarto agudo de miocardio (IAM) y la diabetes mellitus (DM). En las enfermedades infecciosas aumentaron las internaciones, excepto para la sífilis congénita, la tuberculosis pulmonar y las EDA. Entre las enfermedades del embarazo, parto y puerperio se encontró un incremento de la hipertensión arterial (HTA), DM e infección urinaria y una disminución de la preeclampsia y la eclampsia (cuadro 1).

**FIGURA 1.**
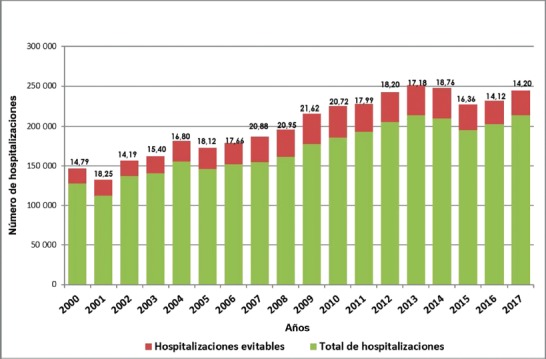
Distribución de hospitalizaciones totales y evitables según el año en Paraguay, 2000-2017

En la figura 1 se ilustra el total de hospitalizaciones y el porcentaje de HCSCA según el año de estudio. Se observa que los porcentajes aumentaron en los años intermedios de la serie (2007-2010) y disminuyeron hacia los últimos años. El porcentaje de HCSCA no mostró diferencias importantes en el tiempo; sin embargo, si se toma en cuenta el año 2009 (cuando se implementó la APS) como punto de corte y se compara el año 2008, previo a la implementación de APS (20,95%) con el año 2017 (14,2%), se aprecia que las HCSCA disminuyeron 6,75%. A pesar de que las internaciones aumentaron, la proporción de HCSCA disminuyó.

**FIGURA 2.**
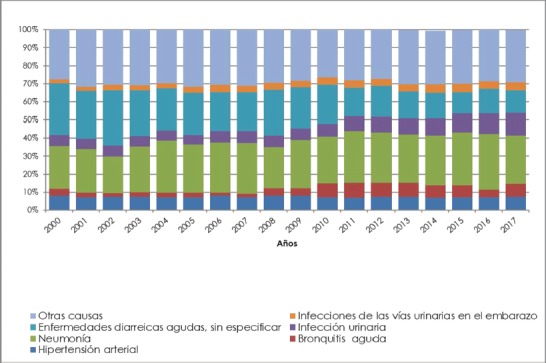
Distribución de hospitalizaciones sensibles a cuidado ambulatorio según el diagnóstico en Paraguay, 2000-2017

Más del 70% de las HCSCA se concentra en seis diagnósticos: neumonía (26,9%), EDA (19,7%), infección urinaria no especificada (7,7%), HTA (7,3%), bronquitis aguda (5,3%) y enfermedad pulmonar obstructiva crónica (EPOC) (4,3%), que suman 71,2% de todas las HCSCA. Este comportamiento es muy estable en todo el período estudiado (figura 2).

**CUADRO 1.** Número y porcentaje de hospitalizaciones evitables por etapas según los diagnósticos

Diagnósticos	Etapa 1 (2000-2008)		Etapa 2 (2009-2017)		Variación porcentual entre las etapas (Δ%)
	N = 226 055	%	N = 313 895	%	
**Enfermedades crónicas no trasmisibles**					
Hipertensión arterial	16 955	7,50	22 806	7,27	0,23
Infarto agudo de miocardio	1 251	0,55	2 350	0,75	-0,20
Insuficiencia cardíaca	3 216	1,42	4 085	1,30	0,12
Enfermedad cerebrovascular	2 436	1,08	2 449	0,78	0,30
Diabetes mellitus	2 859	1,26	4 715	1,50	-0,24
Enfermedad pulmonar obstructiva crónica	12 330	5,45	11 278	3,59	1,86
Asma bronquial	6 444	2,85	6 625	2,11	0,74
Cáncer de cuello uterino	5 073	2,24	5 148	1,64	0,60
**Enfermedades infecciosas**					
Bronquiolitis	7 005	3,10	15 374	4,90	-1,80
Bronquitis aguda	5 882	2,60	21 266	6,77	-4,17
Neumonía	58 017	25,66	87 210	27,78	-2,12
Bronconeumonía	0	0,00	9	0,003	-0,003
Infección urinaria	13 621	6,03	28 155	8,97	-2,94
Sífilis congénita	3 906	1,73	3 810	1,21	0,52
Tuberculosis pulmonar	1 581	0,70	1 819	0,58	0,12
Enfermedad diarreica aguda	55 615	24,60	51 048	16,26	8,34
Infecciones agudas de las vías respiratorias superiores	4 836	2,14	9 903	3,15	-1,01
Celulitis	1 214	0,54	3 715	1,18	-0,64
Otitis media no supurativa y no especificada	2	0,00	151	0,05	-0,05
**Enfermedades del embarazo, parto y puerperio**					
Hipertensión arterial prexistente que complican el embarazo	59	0,03	412	0,13	-0,10
Infecciones de las vías urinarias en el embarazo	7 101	3,14	13 166	4,19	-1,05
Diabetes mellitus en el embarazo	287	0,13	597	0,19	-0,06
Enfermedades infecciosas clasificadas en otra parte, pero que complican el embarazo	2 406	1,06	2 025	0,65	0,41
Otras enfermedades maternas clasificadas en otra parte, pero que complican el embarazo	3 996	1,77	5 614	1,73	0,04
Preeclampsia sin hipertensión crónica	1	0,00	24	0,01	-0,01
Enfermedades virales congénitas	2	0,00	5	0.001	-0,001
Desnutrición en el embarazo	22	0,01	6	0.002	0,008
Edema y proteinuria gestacionales inducida por el embarazo sin hipertensión	21	0,01	13	0.004	0,006
Preeclampsia	5 712	2,53	5 306	1,69	0,84s
Eclampsia	1 318	0,58	869	0,28	0,30
Hipertensión materna no especificada	1 943	0,86	1 446	0,46	0,40
Hipertensión gestacional	323	0,14	1 724	0,14	0,00
**Otros diagnósticos de hospitalización evitable**					
Parasitosis intestinal	19	0,01	70	0,02	-0,01
Anemia ferropénica	22	0,01	185	0,06	-0,05
Desnutrición	323	0,14	118	0,04	0,10
Gastritis	257	0,11	400	0,13	-0,02

***Fuente:*** Ministerio de Salud Pública y Bienestar Social/Dirección General de Información Estratégica en Salud de Paraguay (MSPBS/DIGIES), Subsistema de Egreso Hospitalario (SEGHOSP). Elaborado por los autores.

***Fuente***: Ministerio de Salud Pública y Bienestar Social/Dirección General de Información Estratégica en Salud de Paraguay (MSPBS/DIGIES), Subsistema de Egreso Hospitalario (SEGHOSP). Elaborado por los autores.

**FIGURA 3.**
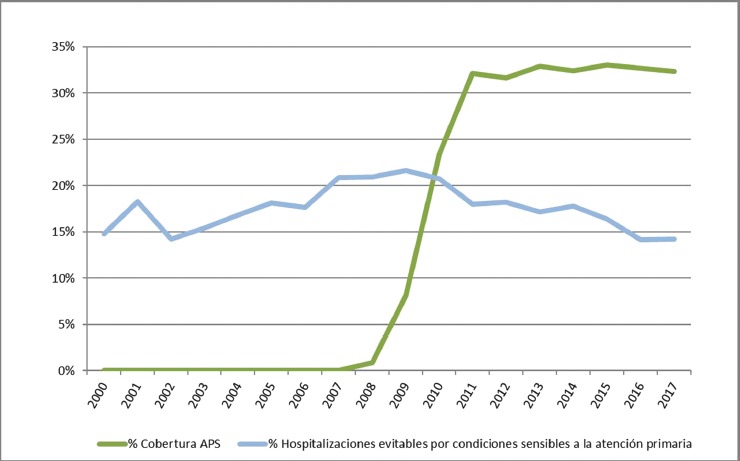
Distribución de hospitalizaciones por condiciones sensibles al cuidado ambulatorio según la cobertura territorial por las unidades de salud familiar en Paraguay, 2000-2017

***Fuente***: Ministerio de Salud Pública y Bienestar Social/Dirección General de Información Estratégica en Salud de Paraguay (MSPBS/DIGIES), Subsistema de Egreso Hospitalario (SEGHOSP). Elaborado por los autores.

El porcentaje de HCSCA tiende a aumentar entre los años 2000 y 2008, y a partir del 2009 disminuye a medida que aumenta la cobertura territorial con USF. A finales del 2008, se implementa la estrategia de APS con 13 USF que cubrían una población de 50 784 habitantes. La creación de nuevas USF fue en aumento, así como también las áreas de responsabilidad; para el 2017 había 801 USF y 2 247 528 habitantes en las áreas de responsabilidad territorial asignada, que correspondía a una cobertura de 32% (figura 3).

**FIGURA 4.**
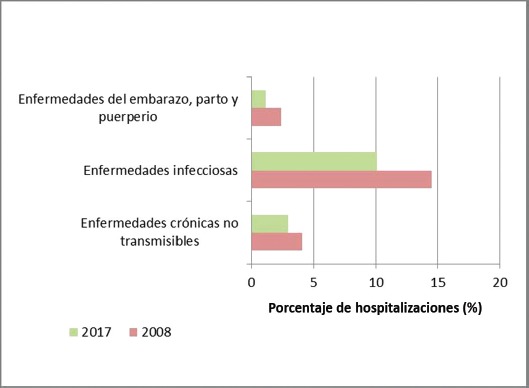
Porcentaje de hospitalizaciones en cada etapa según las categorías diagnósticas en Paraguay, 2008 y 2017

En la figura 4 se comparan los porcentajes de HCSCA, agrupadas por categorías, correspondientes a los años finales de cada etapa (2008 y 2017); se observa una disminución del número de hospitalizaciones en todas las categorías.

## DISCUSIÓN

La APS genera la oportunidad de mejorar los indicadores de salud mediante acciones como la promoción de salud, el diagnóstico y el tratamiento oportuno, la vigilancia estrecha, el saneamiento básico, además de la rehabilitación y la resolución de padecimientos con bajo nivel de complejidad (15). Lo anterior puede explicar el descenso de las hospitalizaciones en el período posterior a la implementación de la estrategia de APS. Si bien el descenso constatado fue de solo 0,1% puede considerarse un resultado importante, dado el gran número de factores que influyen en este índice.

Los resultados superan los reportados por México en el período 2005-2014, donde se informó un 12,4% de HCSCA y se observó, igual que en Paraguay, un descenso a partir del año 2010 (16). En Ecuador, el porcentaje promedio de HCSCA fue de 11,15% (17). Por el contrario, Bogotá reportó cifras superiores a las nuestras, al publicar un valor de 22,5% en cinco hospitales de esta urbe entre los años 2006 y 2008 (11), con una muestra representativa de 263 917 egresos hospitalarios generados entre los años 2006 y 2008 en cinco hospitales de la ciudad.

***Fuente***: Ministerio de Salud Pública y Bienestar Social/Dirección General de Información Estratégica en Salud de Paraguay (MSPBS/DIGIES), Subsistema de Egreso Hospitalario (SEGHOSP). Elaborado por los autores.

Hay particularidades que definen que la APS se asocie a un menor riesgo de HCSCA. Una muy importante es la cobertura territorial de las USF, que para el 2017 era de 32%, por lo que se sugiere orientar las políticas de salud a la profundización de la APS mediante la ampliación del número de proveedores, mayor accesibilidad a los servicios y aumento de la disponibilidad horaria (18). Deben fortalecerse también la continuidad de atención, la APS basada en la familia y orientada a la comunidad y el aumento de prácticas preventivas (1).

Para alcanzar mejores resultados de salud, se ha de contar con recursos humanos resolutivos y polivalentes capaces de abordar todos los problemas de salud comunes en la población asignada (19). Al respecto, hoy en día 709 USF cuentan con médicos, de los cuales solo 126 son especialistas en medicina familiar. Esta situación, junto con la limitada cobertura territorial en el país, podría influir en la discreta disminución de las HCSCA. La resolutividad de los recursos humanos en APS se refuerza con las dotaciones de equipamiento y tecnología adecuados, de modo que los problemas de salud comunes sean diagnosticados, tratados y controlados de manera adecuada en el primer nivel de atención (20).

Un estudio español encontró 13% de HCSCA, con las enfermedades de las vías respiratorias bajas como la primera causa de hospitalización con independencia de la edad (21). Resultados similares se encontraron en la presente investigación. Otro estudio español en pacientes mayores de 65 años señaló que, en 93,1% de los casos, las HCSCA fueron ocasionadas por HTA, insuficiencia cardíaca y neumonía (22). En nuestra serie, la HTA resultó ser la patología crónica que más HCSCA ocasionó en ambas etapas, con ligera tendencia a la disminución en la segunda etapa. La HTA posee una distribución mundial, y sobre ella inciden factores de índole económico, social, cultural, ambiental y étnico. La prevalencia es de 20-30% en la población mayor de 18 años y ha continuado su ascenso asociada a estilos de vida no saludables y desatención de las enfermedades cardiovasculares, cerebrales y renales, lo que ocasiona una disminución significativa de los años de vida y aceleración de la mortalidad (23). Los profesionales de la salud, en especial los de APS, pueden influir sobre muchos de estos factores. Para ello, requieren desarrollar acciones de intervención de salud enfocadas a evitar la aparición de la enfermedad (promoción de salud) y evitar la progresión de la enfermedad mediante el diagnóstico temprano al realizar pesquisas activas, tratamiento oportuno y seguimiento del paciente (10).

En Ecuador, en el año 2014, se detectó que las patologías en las que se vio un ascenso paulatino de su tasa de hospitalización durante los años 2002-2012 fueron diarrea de presunto origen infeccioso, neumonía, infecciones del tracto urinario y diabetes (17).

En México, un estudio de HCSCA que abarcó tres quinquenios encontró que la DM, las gastroenteritis y otras enfermedades de las vías respiratorias inferiores fueron las de mayor porcentaje, y llegaban a constituir casi la mitad de las HCSCA en cada quinquenio (24).

Los autores aprecian que las afecciones de origen infeccioso generan una carga hospitalaria que podría evitarse con un diagnóstico temprano y un manejo adecuado en la comunidad. Una estrategia efectiva para modificar esta realidad sería fortalecer la capacitación de los médicos de APS y crear guías de la práctica clínica que estandaricen la atención brindada para estas y el resto de las condiciones sensibles al cuidado ambulatorio. La disminución de las HCSCA por enfermedades crónicas en la etapa 2 fue discreta para casi todas las causas consideradas dentro de este grupo y más notable en la EPOC, el asma bronquial (AB) y el cáncer de cuello uterino. Los autores consideran que esto puede ser reflejo del diagnóstico temprano del cáncer a través de su pesquisa activa y el manejo oportuno y adecuado de la crisis aguda de asma y sus complicaciones. Para el caso de la EPOC, la reducción puede estar influenciada por la labor educativa del ESF en relación con el hábito tabáquico y sus consecuencias.

Entre las enfermedades infecciosas, se encontró una disminución de más de 8% en las EDA luego de la implementación de la APS. Las EDA constituyen un problema de salud pública en todo el mundo, y está determinado por deficientes condiciones de vivienda, falta de acceso a agua potable y segura y a saneamiento básico e higiene, bajos ingresos, educación deficiente y barreras de acceso a los servicios de salud en general y, en especial, a la atención primaria en salud (25). Lo anterior justifica que la implementación de las USF impacte de manera más importante en estas enfermedades. Las acciones de promoción, prevención, diagnóstico temprano y tratamiento oportuno, inherentes a la APS, resultan muy eficaces en el abordaje de las EDA.

Se debe tener precaución al interpretar las tasas de HCSCA, ya que valoran un conjunto de intervenciones e interacciones dentro del sistema sanitario y no solo dependientes del equipo de salud (17). Factores externos al desempeño de las USF pueden influir en este resultado, como el acceso oportuno a servicios de salud de primer nivel (elemento que consideramos relevante en la interpretación de los datos), la escasa formación de posgrado (especialización) del recurso humano que labora en ese nivel y la falta de disponibilidad y acceso a terapias y métodos diagnósticos adecuados.

Entre las limitaciones de esta investigación, puede mencionarse que la fuente de datos no fue la misma en todos los años de la serie. Antes del 2011, la información era recogida en el departamento de bioestadísticas del MSPBS; así se inició la estandarización y la informatización de los formularios de egresos hospitalarios en el 2011, con la creación del SEGHOSP. Por otro lado, hasta la fecha, solo los datos hasta el año 2013 se encuentran correctamente cerrados y con control de consistencia de las principales variables. Tampoco se dispone de un grupo control para la evaluación de las HCSCA antes y después de la implementación de la APS, lo cual sería la forma ideal de hacer la evaluación del impacto del programa en el contexto de Paraguay.

Se concluye que el impacto en las HCSCA se ha visto limitado por razones estructurales de cobertura y acceso poblacional al primer nivel de atención. No obstante, se encontró una reducción de las HCSCA en casi todas las enfermedades crónicas, más marcada en la EPOC, el AB y el cáncer de cuello uterino. Se observó un incremento de las HCSCA en casi todas las enfermedades infecciosas, excepto en la sífilis congénita, la tuberculosis pulmonar y las EDA; es en estas últimas donde se constata una mayor reducción. Se propone profundizar en la temática mediante nuevos estudios, en la medida que se fortalezca la APS en el territorio.

### Agradecimientos

Los autores agradecen a Damián Sedliak y a Aníbal Lezcano.

### Contribución de los autores

Todos los autores concibieron el estudio original, recolectaron y analizaron los datos, y escribieron y revisaron la versión final.

### Declaración

Las opiniones expresadas en este manuscrito son responsabilidad del autor y no reflejan necesariamente los criterios ni la política de la *RPSP/PAJPH* y/o de la OPS.
